# Heavy Tails and the Shape of Modified Numerals

**DOI:** 10.1111/cogs.13176

**Published:** 2022-07-13

**Authors:** Fausto Carcassi, Jakub Szymanik

**Affiliations:** ^1^ Seminar für Sprachwissenschaft University of Tuebingen Tuebingen Germany; ^2^ Institute for Logic, Language and Computation Universiteit van Amsterdam

**Keywords:** Modified numerals, Rational speech act

## Abstract

The pattern of implicatures of the modified numeral “more than *n*” depends on the roundness of *n*. Cummins et al. (2012) present experimental evidence for the relation between roundness and implicature patterns and propose a pragmatic account of the phenomenon. More recently, Hesse and Benz (2020) present more extensive evidence showing that implicatures also depend on the magnitude of *n* and propose a novel explanation based on the approximate number system (Dehaene, 1999). Despite the wealth of experimental data, no formal account has yet been proposed to characterize the full posterior distribution over numbers of a listener after hearing “more than *n*.” We develop one such account within the Rational Speech Act framework, quantitatively reconstructing the pragmatic reasoning of a rational listener. We argue that world knowledge about the distribution of the true quantity has a substantial impact on the information conveyed by the modified numeral. We show that our pragmatic account in combination with a heavy‐tailed model of the participants' prior correctly predicts various features of the experimental data from Hesse and Benz (2020).

## 1 Introduction

Traditional pragmatics mostly limited itself to *qualitative* accounts of implicatures, in which an utterance in a context implicates some propositions, excluding or including some possible world states. For instance, “most cats knit” implicates that it is not the case that all cats knit. In the past 20 years, new experimental and statistical methods have been applied to capture subtler patterns in speaker behavior (see, e.g., Cummins & Katsos, 2019, for an overview). In particular, the development of Bayesian cognitive models of pragmatic language use and sophisticated experimental designs have allowed researchers to test more fine‐grained hypotheses about graded notions of implicature (Franke & Bergen, 2020).^1.^ In this picture, rather than a qualitative difference between states pragmatically compatible or incompatible with an utterance, a pragmatic listener has a full prior over states which is updated after receiving the utterance. The semantic and pragmatic content of the utterance contributes to the listener's estimated probability of each possible state, allowing for a graded and quantitative notion of compatibility between an utterance and a possible state.

As a case study in this approach to pragmatics, in this paper we look at modified numerals, that is, expressions such as “more than 3.” Modified numerals usually convey information about the cardinality of the intersection of two sets. For instance, “about 4 Frenchmen yawn” conveys that the cardinality of the intersection of the set of Frenchmen and the set of yawning things is not far from 4. Examples of modified numerals are “at least 4,” “exactly 1,” and “more than 3.” In this paper, we focus on the latter expression: “more than *n*” (for some integer *n*). We develop an account of the shape of the posterior distribution over numbers of a language user upon hearing an expression containing a modified numeral.

While the meaning of “more than *n*” might at first appear straightforward, the usage of the expression poses several puzzles. First, as noticed already in Krifka (1999) and experimentally confirmed in Geurts (2010), the standard Horn account is at odds with the behavior of modified numerals. Specifically, modified numerals do not seem to elicit some of the predicted scalar implicatures, for example, “more than 3” does not seem to implicate “not more than 4.”^2.^ Second, as discussed in Cummins et al. (2012), the implicatures drawn from “more than *n*” are influenced by the *roundness* of *n*. For instance, “more than 10” seems to implicate “not more than 20,” but not “not more than 13.” This might be a consequence of “20” being more round than “13.” Third, as discussed in Hesse and Benz (2020), the range of numbers for which “more than *n*” is used is in some contexts influenced by the *magnitude* of *n*. Specifically, the greater the magnitude of *n*, the greater the range of numbers above *n* to which “more than *n*” still probably applies. For instance, “*m* is more than 10” prima facie would not be used when m=1,010, but “*m* is more than 25,000” seems appropriate for m=26,000, although the difference between *m* and *n* is the same in the two cases.

**Fig. 1:**
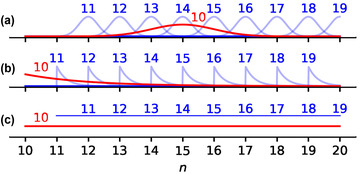
Three possible usage distribution patterns for modified numerals “more than 10” to “more than 19” (the distributions are over integers albeit showed as continuous for ease of visualization). Each line corresponds to a different utterance of the form “more than *n*” and shows the posterior distribution of a listener after hearing the signal. The *n* is shown above each distribution's peak. Blue lines are used for the numerals of the lowest roundness level, and the red line is used for “more than 10,” which is more round.

These three puzzles constitute qualitative patterns in the implicatures induced by various modified numerals. However, they are all downstream from the posterior distribution over numbers induced in a listener after hearing “more than *n*,” formally p(·|Speakeruttered’morethann’), which we abbreviate to p(·|MTn). In other words, p(·|MTn) describes the probability that the listener attributes to *m* being each number after hearing “*m* is more than *n*” (with *n* known and *m* unknown). p(·|MTn) encodes the pattern of implicatures, as well as their dependence on the roundness and magnitude of the modified numeral.

p(·|MTn) could prima facie take various shapes.^3.^ We consider three for illustration. First, p(·|MTn) could approximate a normal distribution with mean *m*, whose mean could depend on the roundness and magnitude of the modified numeral (option *a* in Fig. 1). Second, p(·|MTn) could approximate an exponential distribution shifted to start at *n*, with a variance that again depends on the roundness and magnitude of *n* (option *b* in Fig. 1). Third, p(·|MTn) could look like a uniform distribution from *n* to some value that could depend on the roundness and magnitude of *n* (option *c* in Fig. 1).^4.^

The three categorical implicature effects we discussed are compatible with all three options from Fig. 1. First, for all three options “more than *n*” can in general cover n+2, implying that “not more than n+1” is not implicated. Second, in all options “more than 10” can behave differently from “more than *n*”, with *n* less round than 10. Lastly, in all three options the relevant parameters can be sensitive to the magnitude of *n*. In sum, a full account of the information conveyed by a modified numeral to a *pragmatic* listener should therefore characterize the full posterior distribution of a listener upon hearing a modified numeral.

Little work has been done to predict, characterize, or describe this distribution as a result of pragmatic reasoning.^5.^ The main aim of this paper is to propose a quantitative model of p(·|MTn) which can account for the previously discussed qualitative patterns as well as experimental data in previous literature and that can generate novel empirical predictions. We find that a small modification to a popular computational model of pragmatic reasoning, the Rational Speech Act (RSA) framework (Franke & Jäger, 2016), can account for the observed shape of p(·|MTn) for a variety of levels of roundness and magnitudes. Moreover, we find that participants' world knowledge about the involved quantities can in large part explain the effect of magnitude on p(·|MTn).

## 2 Previous literature and data

### 2.1 Granularity‐based approaches

Early work on “more than *n*” focused on roundness as a possible factor to explain the unusual implicature patterns discussed above. The concept of roundness can be analyzed in terms of the concept of scale. Scales consist of the set of multiples of certain numbers, for example, 5, 10, 50, 100, which are particularly cognitively simple.^6.^ One scale is finer grained than another if it divides the number scale into points that are closer together. The roundness of a numeral can then be thought of as the level of the least finely grained scale to which the numeral belo. As an example, consider 30 and 200. Two hundred belongs to many scales—e.g., the ones containing the multiples of 1, of 2, of 10—but the finest grained scale it belongs to is arguably the scale of multiples of 100. On the other hand, the finest grained scale 30 belongs to is that of the multiples of 10. Two hundred is therefore more round than 30 because the former belongs to a more coarse‐grained scale than the finest grained scale where the latter figures.

Cummins et al. (2012) argue that roundness plays a role in the pattern of implicatures of modified numerals.^7.^ For instance, “more than 1,000” lacks the implicature “not more than 1,001.” In order for the implicature to be calculated, the listener would have to assume that, had the speaker observed, for example, 1,002, they would have said “more than 1,001.” However, 1,000 is rounder than 1,001, and therefore uttering 1,001 comes at an additional cognitive cost compared to 1,000. For the speaker, the additional cognitive cost is too great for the little additional information conveyed by uttering “more than 1,001.” The listener cannot, therefore, infer that the speaker would have said “more than 1,001” had they observed a state (e.g., 1002) for which “more than 1,001” would have been only slightly more informative than “more than 1,000.”

Crucially, Cummins et al. (2012) note that the same argument does not apply when the implicated sentence contains a numeral at the same or greater roundness level as *n*. For instance, after observing 2,300 a speaker would rather utter “more than 2,000” than “more than 1,000,” everything else being equal, because the two involved numerals are cognitively equally costly, but the former utterance is more informative. Therefore, the listener would have reason to infer that if the speaker uttered “more than 1,000,” they did not observe a state for which “more than 2,000” applies. More generally, “more than *n*” should generate a scalar implicature to “not more than *m*,” where *m* is the next numeral at the same roundness level of *n*. Moreover, Cummins et al. (2012) point out that, for similar reasons, “more than *n*” should also implicate “not more than *m*” for any *m* at a higher roundness level than *n*. For instance, “more than 90” is predicted to implicate “not more than 100.”

Based on these arguments, Cummins et al. (2012) make two experimental predictions. First, the rounder the *n*, the higher responders' estimates will be compared to *n*. Second, in the range condition, typical estimates will be of the form “n+1 to *m*,” where *m* is the value after *n* with the same granularity as *n* or higher.

Cummins et al. (2012) then present an experiment to test the two predictions. Participants (n=1,200) were presented with 16 contexts. The following is one such context (varying by condition as indicated):

**Information** A newspaper reported the following. “[Numerical expression] people attended the public meeting about the new highway construction project.”

**Question** Based on reading this, how many people do you think attended the meeting?

Between         and         people attended [range condition].

        people attended [single number condition].

The numerical expression included a quantifier (either “more than” or “at least *n*”) and a numeral belonging to one of three levels of granularity (multiples of 100, multiples of 10 but not 100, and non‐round such as 93).

Overall, the results confirmed the two experimental predictions. The range of interpretation increases with the roundness of the numeral. Moreover, most responses in the range condition were as predicted. For instance, “more than 100” typically conveyed an upper bound at 150.

### 2.2 Hesse and Benz (2020)

Hesse and Benz (2020) consider two empirical predictions from Cummins et al. (2012). First, the rounder the *n* in “more than *n*,” the wider the range of potential values. Second, the rounder the *n*, the further away from *n* will be the single most likely value. In a series of experiments, Hesse and Benz (2020) test these two predictions for a wider range of numerals than Cummins et al. (2012) and find that they are not borne out. Moreover, new patterns in the interpretation of modified numerals emerge from the data.

The first experiment identifies some domains for which participants do not have strong prior beliefs as to the size of the involved numerals. In such contexts, prior belief does not play a strong role in the estimation of the numerals, and therefore the effect of roundness and magnitude can be isolated from other prior factors. Four such domains are identified: petition signatures, audience size at a music concert, votes in an election, and spectators at a sporting event. The second experiment is a replication of the experiment in Cummins et al. (2012) for a wider range of numerals at four different roundness levels (Coarse: 100, 200, medium roundness: 50, 150, low roundness: 90, 110, 130, and unround: 93). Results do not corroborate the two predictions based on Cummins et al. (2012): the (median) distance between *n* and the guessed number in the single number condition does not increase the rounder the *n* is, and neither does the (median) range in the range condition. In the third experiment, numerals of a wider range of magnitudes are tested (20, 30, 40, 60, 70, 80, 120, 140, 160, 170, 180, and 190) and only the four contexts identified in experiment 1 are used. In the combined data from the second and third experiments (as well as in the data from the second experiment alone), magnitude is a stronger predictor of the range of produced values than roundness.

**Fig. 2:**
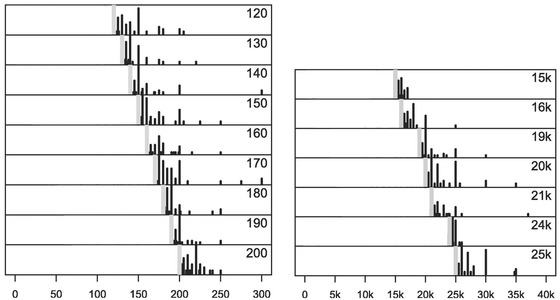
Some participants' responses from the single numbers condition in experiment 2 (left, low magnitude) and 3 (right, large magnitude) in Hesse and Benz (2020). Responses for “more than *n*” are shown on the right side of the gray line, and *n* is shown on the top right of each subplot.

The second and third experiments in Hesse and Benz (2020) fail to find evidence for two of the effects predicted by Cummins et al. (2012). However, two novel patterns emerge. First, the median numbers in the single number condition are a constant distance of 10 above the modified numeral. Second, participants tend to guess numbers with an upper bound located at the next round number above the modified numeral. For instance, when presented with “more than 120,” “more than 130,” or “more than 140” participants tend to guess numbers up to 150 (a round numeral). This produces a “squeezing” effect for modified numerals immediately below a round number. Both patterns can be observed in the left plot in Fig. 2.

In the fourth and last experiment, Hesse and Benz (2020) focus on larger numerals. They administer the same task, with six contexts (number of signatures on a petition, size of the audience at a music concert, turnout at an election, number of spectators at a sporting event, size of a meeting, and budget for a reception) and larger numerals (1k, 1.1k, 1.4k, 15k, 16k, 19k, 20k, 21k, 24k, 25k). Like in the previous experiments, greater roundness does not per se cause a greater range of guessed numbers or wider ranges in the range condition. Moreover, the two new patterns noticed in the previous experiments persist, but scale proportionally to the magnitude of the involved numerals. While in the 1–100 range the median guessed number was around 10 above the modified numeral, in the range of thousands it is 100 above, and in the range of tens of thousands it is 1,000 above. While the upper boundaries participants tend to select are at the roundness level of multiples of 50 or 100 in the 100 interval, they are multiples of, for example, 500 in the 1,000 range and multiples of 5k in the tens of thousands range. Both effects can be seen in the right plot in Fig. 2.

Hesse and Benz (2020) also give a characterization of their data in terms of a boundary function, and they propose an explanation for the fact that larger *n*s lead to guesses with a proportionally greater variance. The explanation relies on the Approximate Number System (ANS), namely the cognitive mechanism that underlies the approximate perception of magnitudes. When using the ANS, numbers are not encoded precisely, but rather as distributions over numbers. Moreover, the variance of this distribution increases with the magnitude of the number. Hesse and Benz (2020) argue that, as the modified numeral gets larger, participants will associate the numeral with increasingly wide distributions, and therefore the spread of their guessed numbers will also increase.

The account developed in Hesse and Benz (2020) paints a clear picture of the patterns in production for modified numerals of the form “more than *n*” and “less than *n*.” However, the paper does not give a full model of the way a listener calculates a posterior over numbers. In order to evaluate their proposal quantitatively, more detail would be needed for the implementation, specifically concerning the relation between the ANS component of their account and roundness. For instance, a bare ANS account alone leaves unexplained why participants tend to produce signals at higher levels of roundness when dealing with larger numbers, rather than simply producing from a distribution with greater variance. Among the 1,000 numerals guessed in the fourth experiment for n≥15,000, only 88 were at a level of roundness lower than 500. As we argue in the next section, a simple model of recursive mindreading can explain these various patterns in the data in a unified manner.

## 3 A Bayesian model for “More Than”

### 3.1 The rational speech act framework

In the following, we propose a characterization of p(·|MTn) based on the RSA framework, an approach to modeling the process of recursive mindreading that lies behind the pragmatic interpretation or production of utterances (Frank and Goodman, 2012; Franke & Jäger, 2016; Goodman & Frank, 2016). RSA models usually start with a pragmatic listener who interprets utterances based on the simulated behavior of a pragmatic speaker. The pragmatic speaker in turn given an observation tends to choose the most useful utterance for a literal listener who interprets it based solely on its literal meaning. We will first explain the simplest type of the RSA model and then a modification that will be useful to model modified numerals.

The simplest RSA model starts with a set of utterances *u* and a set of possible states *s*. The meaning of each utterance can be encoded for our purposes as the set of those states that verify the utterance. The pragmatic listener *L*_1_ receives an utterance *u* and calculates a posterior over states by Bayesian update, combining their prior over states with the probability that the pragmatic speaker *S*_1_ would have produced the utterance given each state:

(1) $$p_{L_{1}}\left(s|u\right)\propto p_{L_{1}}\left(s\right)p_{S_{1}}\left(u|s\right).$$

The pragmatic speaker in turn observes a state and produces an utterance with a probability that depends on a cost‐related salience utterance prior weighted by parameter β (see chapt. 3 in Scontras, Tessler, & Franke, 2018):

(2) $$p\left(u;C\right)\propto \exp\left(-\beta c(u)\right)$$

and on the utility U(u|s) for a literal listener *L*_0_ given the state:

(3) $$p_{S_{1}}\left(u|s\right)\propto \exp\left(\alpha U\left(u|s\right)\right)p\left(u;C\right),$$

where α is the speaker's rationality parameter: the higher the value of α, the more the speaker's distribution will be peaked at the most useful utterances. The utility U(u|s) is the negative surprisal of the state given the utterance so that the speaker favors utterances that make the state less surprising for the literal listener:

(4) $$U\left(u|s\right)=\log(p_{L_{0}}\left(s|u\right)).$$

Finally, the probability that literal listener *L*_0_ attributes to each state given an utterance is simply 0 if the utterance is not verified by the state and proportional to the prior for the state otherwise:

(5) $$p_{L_{0}}\left(s|u\right)\propto \left\{\begin{array}{cc} p_{L_{0}}\left(s\right) & \text{if}\;s\;\text{verifies}\;u\\ 0 & \text{otherwise} \end{array}\right..$$

**Fig. 3:**
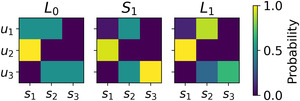
Simple RSA model with three possible utterances *u* (*y*‐axis) and three states *s* (*x*‐axis). *L*_1_ calculates a scalar implicature for utterances *u*_1_ and *u*_2_ (α=4). The left, central, and right plots correspond to *L*_0_, *S*_1_, and *L*_1_ respectively. The color indicates the probability of guessing a state given a signal for *L*_0_ and *L*_1_, and the probability of producing a signal given a state for *S*_1_.

Fig. 3 shows *L*_0_, *S*_1_, and *L*_1_ in this simple RSA model. The crucial phenomenon that can be observed in Fig. 3 is that *L*_1_ calculates a scalar implicature: although utterance *u*_1_ is, in its literal sense, compatible with both *s*_1_ and *s*_2_, *S*_1_ tends to produce *u*_1_ mostly for *s*_2_, because when *s*_1_ is observed *S*_1_ tends to use the more useful signal *u*_2_. Therefore, when hearing *u*_1_ *L*_1_ is more likely to guess *s*_2_.

### 3.2 An RSA model for modified numerals

We start by introducing two simple changes to the basic RSA model above to get a first approximation of the experimental data in Hesse and Benz (2020). First, we let utterance cost depend on the roundness level in a way consistent with Hesse and Benz (2020)'s measure of roundness, itself based on the measure in Cummins et al. (2012) and inspired by Jansen and Pollmann (2001). Specifically, we calculate the cost as the inverse rank of roundness, in the following way:

**Table:** 

Finest grained scale	Cost c(n)	Coarsest grained scale	Cost c(n)
1,000	0	50	4
500	1	10	5
200	2	5	6
100	3	1	7

**Fig. 4:**
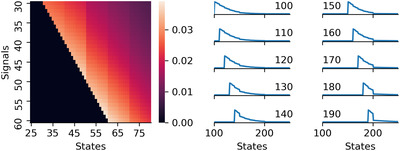
Left: Listener's posterior probability p(·|MTn) over numbers given each signal. The squeezing effect can be visualized as the increasing concentration of posterior mass approaching round numbers from below (α=7,β=1). Right: p(·|MTn) for *n* at intervals of 10. The squeezing effect—the posterior distribution concentrating between *n* and the closest round number above—is particularly clear for n=140,190.

For instance, according to this measure 3,000—whose greatest divisor in the table is 1,000—gets cost 0, while 350—whose greatest divisor in the table is 50—gets cost 4. The relation between roundness level and cognitive cost is confirmed (for a different but related definition of roundness) by Solt, Cummins, and Palmović (2017), which gives experimental evidence that the level of granularity influences the cognitive complexity of the expressions. Second, we specify a prior for both the literal listener, $p_{L_{0}}$, and the pragmatic listener, $p_{L_{1}}$. For simplicity, we assume that the two priors are identical. This corresponds to the default assumption that the literal listener assumes that the pragmatic speaker has an accurate representation of the listener's prior. We provisionally assume for purposes of exposition that this prior has a geometric distribution with parameter k=0.007, and we revise this prior in a more principled manner in the next section. The results of these two changes for small *n* can be seen in Fig. 4 for numerals between 100 and 190.^8.^

Some crucial features of the data are predicted by adding granularity‐dependent cost and a prior at the *L*_0_ and *L*_1_ levels. First, as observed in the data in Hesse and Benz (2020), the distribution for “more than *n*” resembles option *b* in Fig. 1. The reason for this, which as far as we are aware has not been discussed in the literature, is an accumulation of very weak scalar implicatures.^9.^ If the listener hears “more than *n*” and considers whether the true state is *m*, then they reason that for all *j* such that n<j<m, the speaker had to choose to not utter *j*, since all expressions “more than *j*” would also be true. Therefore, the greater the number of *j*s, the less plausible it is that the true state is *m*.

A second feature in the data that our model correctly predicts is the effect of the numeral's roundness on the distribution of guesses. In partial agreement with Cummins et al. (2012), some round numbers have a greater variance than less round numbers. For instance, “more than 0” is predicted to have greater variance than “more than 20.” However, contra to Cummins et al. (2012) and consistent with Hesse and Benz (2020), the effect is small for all numbers except 0 in the model.

A third feature in the data captured by our model is the squeezing effect. As the signal approaches a round number from below, the probability mass becomes more concentrated between *n* and the round number. This is clear in the right plot in Fig. 4 for numerals approaching 150 and 200. The effect also exists, but to a lesser extent, for numerals of lower roundness levels. As already described in Cummins et al. (2012), the effect is a consequence of an implicature. If the true number had been higher than a round numeral higher than the one the speaker chose, the speaker would have chosen the higher round numeral instead. Therefore, the number has to be lower than any round numeral above the one actually chosen by the speaker.

**Fig. 5:**
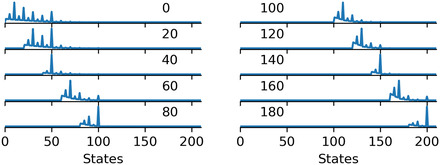
Predicted production probabilities for a simple production model based on an RSA pragmatic listener *L*_1_ (α=7,ρ=3). The production model correctly describes the participants' tendency to guess a round number above the observed number.

While the simple model of the pragmatic listener can account for some features of the experimental data, it differs in a crucial way from the participants in the experiment. Namely, the RSA pragmatic listener is a pure listener, while the participants were asked to produce a guess, and therefore are also in a certain way speakers. In order to make predictions comparable to the experimental data presented in Hesse and Benz (2020), we also propose a simple way that the listener might use their posterior over states given a modified numeral to give a response in the experiment. In our participant model, listeners tend to produce states that have a high probability, with an additional utterance prior against producing signals with a high cost:

(6) $$p\left(\text{Producing}\;m|u\right)\propto \exp\left(\rho \log(p_{L_{1}}\left(m|u\right))-c\left(m\right)\right),$$

where *u* is the utterance shown to the participant and ρ is the softmax (inverse temperature) parameter, encoding a tendency of the listener to select the signal with the highest posterior probability. This model of participant production reflects the speaker model of pragmatic RSA agents, for a listener who is asked to make a guess as to the world state. Fig. 5 shows the predicted production probabilities for a participant in the single number condition. The predictions resemble the observed data shown in the left plot of Fig. 2, both in terms of which numerals are produced and more generally in terms of the shape of the produced numerals.

### 3.3 The effect of the order of magnitude

While the simple RSA model we presented here correctly captures various features of the experimental data, it does not explain the observed relation between the magnitude of the modified numeral and the range of participants' guesses. Hesse and Benz (2020) propose to explain this relation with a *cognitive* mechanism, the ANS. On the other hand, we propose to explain the effect of magnitude on the observed distribution of guesses based only on participants' world knowledge, as encoded in their prior. We focus on the contexts that Hesse and Benz (2020) use for the production tasks in experiments 2, 3, and 4, which show the effect of magnitude. For these experiments, Hesse and Benz (2020) select those contexts for which participants have a wide range of guesses in experiment 1 (called “weak prior beliefs” in Hesse & Benz, 2020), as opposed to the contexts where guesses cluster together (“strong prior beliefs”). While we agree that two types of priors indeed appear among the contexts in experiment 1, we believe their specific functional shape has a crucial role to play in explaining the effect of magnitude. Namely, we argue in this section that what Hesse and Benz (2020) call “weak priors” are in fact heavy‐tailed distributions with a special property which we define below. This will provide a precise explanation of the connection between contexts with “weak prior beliefs” and the effect of magnitude.

Before discussing the case at hand, it is worth briefly reviewing the different possible behaviors for the tails of a distribution. Informally, the tail of a distribution is the part of the distribution that describes values far away from the distribution's mean.^10.^ Tail behavior determines various important aspects of the distribution, some of which we return to below. Nonetheless, a fundamental distinction can be drawn between distributions with heavy or light tails. A random variable *X* is said to be heavy‐tailed if and only if for any μ>0,

(7) $$\underset{x\to \infty }{\lim\;\sup}\frac{P(X>x)}{e^{-\mu x}}=\infty .$$

Intuitively, this means that the tail of the distribution decreases slower than that of an exponential distribution. While this might appear like an arbitrary diving line between two groups of distributions, heavy‐ and light‐tailed distributions behave qualitatively different in a number of ways. While values much bigger than the majority of the values are extremely unlikely with light‐tailed distributions, they can be expected with heavy‐tailed distributions.

Our first step in finding the most appropriate prior for the RSA model is to find an appropriate family of priors for the contexts under discussion. We begin approaching the problem of finding the right prior empirically by simply fitting three possible distribution families with positive support on the prior samples from experiment 1 (without modified numerals). We consider three families of distributions with discrete positive support: geometric, zeta, and negative binomial. These three distributions are a natural choice in this context. Among light‐tailed distributions, the negative binomial has a nice interpretation as a compound distribution: it is the distribution of a Poisson‐distributed variable whose λ parameter has a Gamma distribution. In other words, if someone thinks a quantity, for example, the number of people attending a concert, is determined by independent events happening in a certain interval of time with a constant rate (Poisson distributed) but is unsure what this rate is (Gamma distributed), then they give this quantity a negative binomial distribution. The zeta distribution does not have such a straightforward generative characterization, but its heavy‐tail behavior has a nice property, namely a linearly increasing *excess mean function*, to which we return below, that makes it particularly appropriate in the context of the data. Finally, the geometric distribution serves as a “line of demarcation” between the property of interest for us: roughly, all and only those positive discrete distributions whose tail is heavier than a geometric distribution have an increasing mean excess function, since the geometric distribution is *memoryless* (Feller, 1957).

**Fig. 6:**
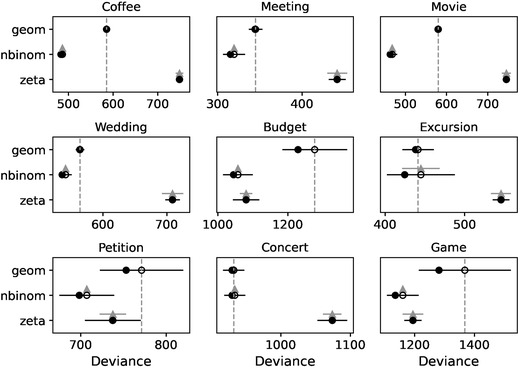
Comparison of three ways of modeling the data from experiment 1 (without numerals): assuming that guesses come from a geometric, a negative binomial, or a zeta distribution. While the former two are light tailed, the zeta is a heavy‐tailed distribution. The empty circles show the approximated leave‐one‐out predictive error on the deviance scale, along with their standard deviation (black error bar). The lower the value, the better the model for the data. Triangles show the difference between a model and the top model, along with its standard error. The observations in some contexts are better modeled with a light‐tailed distribution (e.g., the size of the audience at a music concert), while a heavy‐tailed distribution is equally good in other contexts (e.g., the number of signatures in a petition).

In order to fit the data to each family of distributions, we assume for each family that participants' guesses are independently drawn with the same parameter values and find a posterior distribution over these.^11.^ Fig. 6 shows the results of fitting each of the three distribution families on the prior samples in experiment 1 in Hesse and Benz (2020). In some contexts, the negative binomial distribution shows a better fit to the data than the zeta distribution (Coffee, Meeting, Movie, Wedding, Excursion, Concert), and in other contexts they are approximately equal (Budget, Petition, Game). The geometric distribution is never better than the other distributions for the contexts of interest, namely those with “weak priors” in the terminology of Hesse and Benz (2020).

**Fig. 7:**
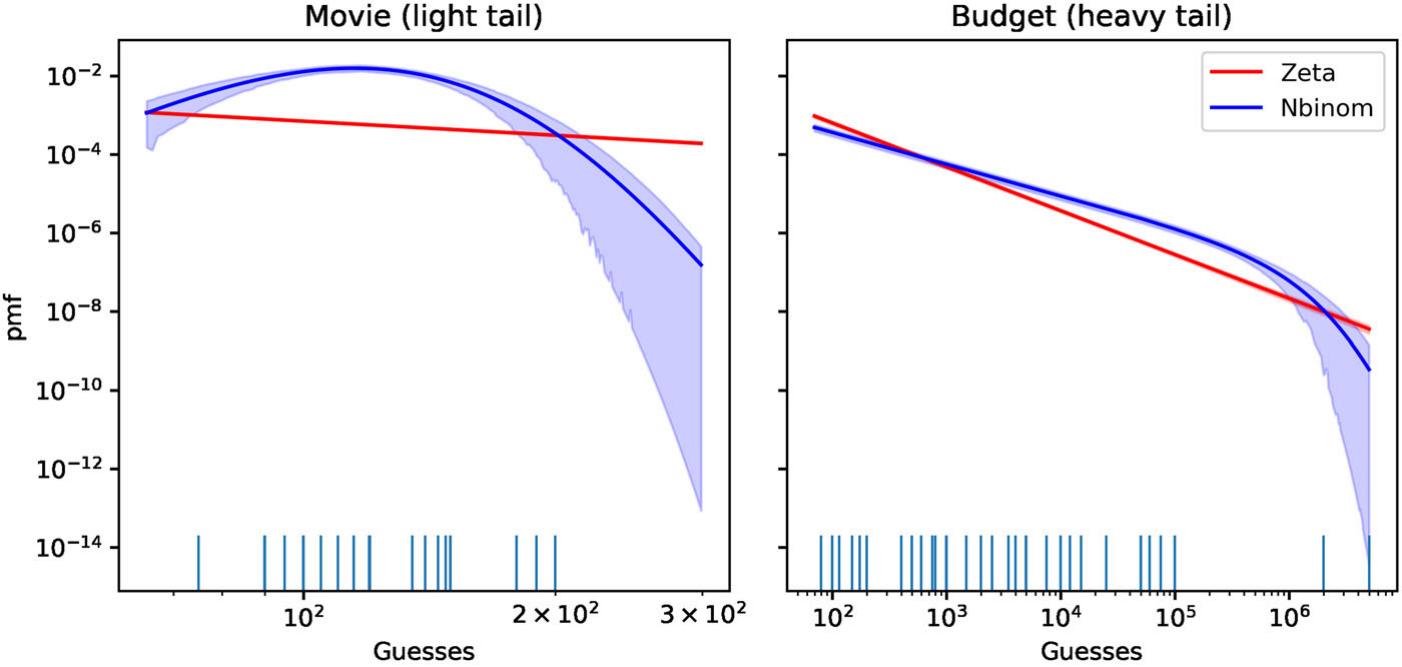
Comparison of the posterior probabilities of the guesses in experiment 1 (without modified numerals) under the zeta distribution and the negative binomial distribution models, for two contexts. The rugplot at the bottom of the plot shows the values guessed by the participants. Note that both the *x* and the *y* axes are in log space. The two models differ in two main ways, as can be seen in the plot.

Fig. 7 paints a clearer picture of the difference in how the zeta and the negative binomial families fit the data, which helps understand how each fails or succeeds. The crucial difference between the two families can be seen in the behavior of the tail. The zeta distribution follows a power law, and therefore its probability mass function^12.^ decreases log‐linearly, while the probability mass function of the negative binomial eventually decays faster than the zeta distribution. Therefore, in the Movie context where observations cluster together fairly closely the zeta distribution cannot do better than the negative binomial for any of the observations. In contrast, in the Budget context where some observations are very large most of the observed data still has a higher probability under the negative binomial, while the most extreme data are more likely under the zeta distribution. In sum, the zeta distribution mostly fails compared to the negative binomial distribution, but even a single extreme enough sample suffices to compensate. This pattern is representative of the other contexts with heavy tails.

It should be noted at this point that there is an asymmetry between the zeta and the negative binomial in terms of how successfully we can expect them to fit the data. If the true participants' prior is light tailed, the data can likely be fit reasonably well with a negative binomial based on its generative characterization discussed above, while a zeta distribution would overestimate the probability of events larger than the ones observed. However, the heavy‐tailed case is more complex. Many naturally occurring heavy‐tailed distributions behave differently in their body and their tails. Therefore, even if the participants' prior is heavy tailed, its body might still not resemble a zeta distribution. Therefore, only considering the zeta distribution underestimates how well the assumption of heavy‐tailedness fits the data. Insofar as we are taking zeta and negative binomial to represent heavy‐ and light‐tailed distributions, respectively, we are therefore giving an unfair advantage to the latter. One option would be to only consider the highest order statistics. However, estimating heavy‐tailedness directly from samples is difficult, especially with few samples.

Fortunately, we can take an alternative route to help restrict the shape of participants' prior, by looking at the data from the remaining experiments. In particular, we consider the observation by Hesse and Benz (2020) that the spread of participant's guesses grows approximately linearly with the magnitude of the modified numeral. If we momentarily put aside the pragmatic phenomena influencing the data, we can think of this observation as a constraint on the prior density *f*. Namely, the constraint that as we lower truncate *f* at higher values, the mean of the resulting distribution grows further away from the lower bound.^13.^ This property can be expressed in terms of the *mean excess function*:

(8) e(x)=E(X−x∣X>x).

To get an intuition for the different ways the mean excess function can behave, compare the two cases of height and wealth. Height has a decreasing mean excess function: if you know someone is taller than 160 cm, you expect them to be roughly 20 cm taller than that. However, if you know someone is taller than 200 cm, you expect them to be at most a few centimeters taller than that. On the other hand, wealth has an increasing mean excess function: if you know someone earns 20k euros yearly, you might expect them to earn just a few thousand euros above that threshold. If someone earns more than 20 million dollars, they probably make at least a few million dollars more. In the context of the experiments in Hesse and Benz (2020), we propose that “weak priors” are in fact best described as distributions with an approximately *linearly increasing* mean excess function. This can be roughly imagined as follows. When truncating the prior distribution at some lower bound *n*, as *n* increases the resulting distribution gets progressively flatter. Moreover, the rate of increase in flatness does not change with *n*.

**Fig. 8:**
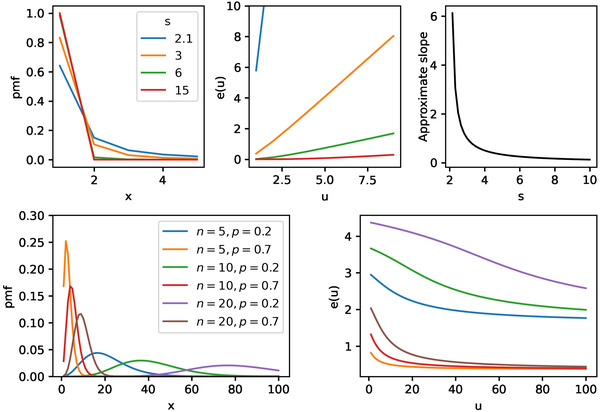
Comparison of mean residual functions for the zeta distribution (top row of plots) and the negative binomial distribution (bottom row of plots). The top left plot shows the probability mass function of the zeta distribution for four parameter values. The top central plot shows the mean residual function *e* for the same parameters. Note that *e* soon approximates a positive linear function, with a slope depending on the zeta's *s* parameter. The relation between *s* and the approximate slope of *e* is shown in the top right plot: as *s* increases, the slope tends towards zero. The bottom left plot shows the probability mass function of the negative binomial distribution for a variety of parameters. The bottom right plot shows the corresponding mean residual functions *e*. Note that, in contrast to the case of the zeta distribution, *e* is decreasing for the negative binomial distribution. The mean residual function of the geometric distribution is simply a constant function, as therefore conceptually serves as the separation between light‐ and heavy‐tailed discrete distributions.

The mean excess function of the zeta distribution increases approximately linearly. Fig. 8 shows a comparison of the mean excess functions of the zeta and the negative binomial. These observations, abstracted from the specific samples that might be influenced by the involved pragmatic phenomena, support the claim that the contexts with “weak” priors in Hesse and Benz (2020) are in fact approximately zeta distributed.^14.^ Therefore, we implement a zeta distribution as the prior in the RSA model presented above.

The strength of RSA in this context is that it allows us to naturally implement this information about the participants' prior distribution in the model. Under the assumption that the prior distribution over states of *L*_0_ and *L*_1_ belongs to the zeta family, the posterior over states of *L*_1_ become

(9) $$L_{1}\left(x|n,s\right)\propto \frac{1}{x^{s}}S_{1}\left(n|x,s\right)$$

(10) $$=\frac{1}{x^{s}}\frac{\zeta {\left(s,n\right)}^{\alpha }e^{-\beta C(n)}}{\sum_{i=1}^{x-1}\left(\zeta {\left(s,i\right)}^{\alpha }e^{-\beta C(i)}\right)}\;\text{with}\;n<x$$

(11) $$\propto \frac{1}{x^{s}\sum_{i=1}^{x-1}\left(\zeta {\left(s,i\right)}^{\alpha }e^{-\beta C(i)}\right)}\;\text{with}\;n<x,$$

where ζ is the Hurwitz zeta function, defined as

(12) $$\zeta \left(s,i\right)=\sum_{n=0}^{\infty }\frac{1}{{\left(n+i\right)}^{s}}.$$

**Fig. 9:**
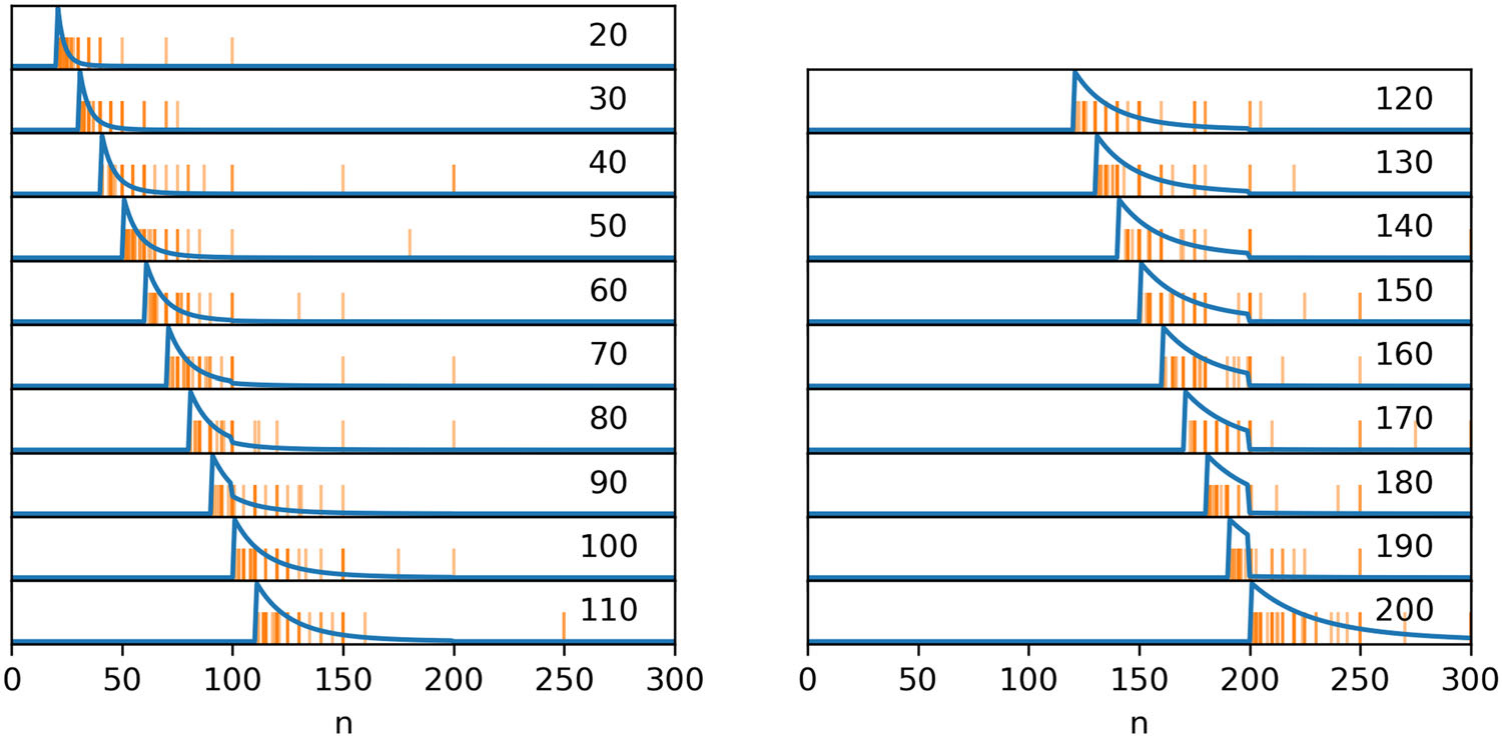
The predicted posterior over numerals after hearing “more than *n*” with a zeta‐distributed prior (s=7,α=0.1,β=5), for small values of *n*. Participants' guesses are shown as vertical orange lines, while the predicted distribution of the guesses is shown with a blue line. Note that the parameters are the same as in Fig. 10.

**Fig. 10:**
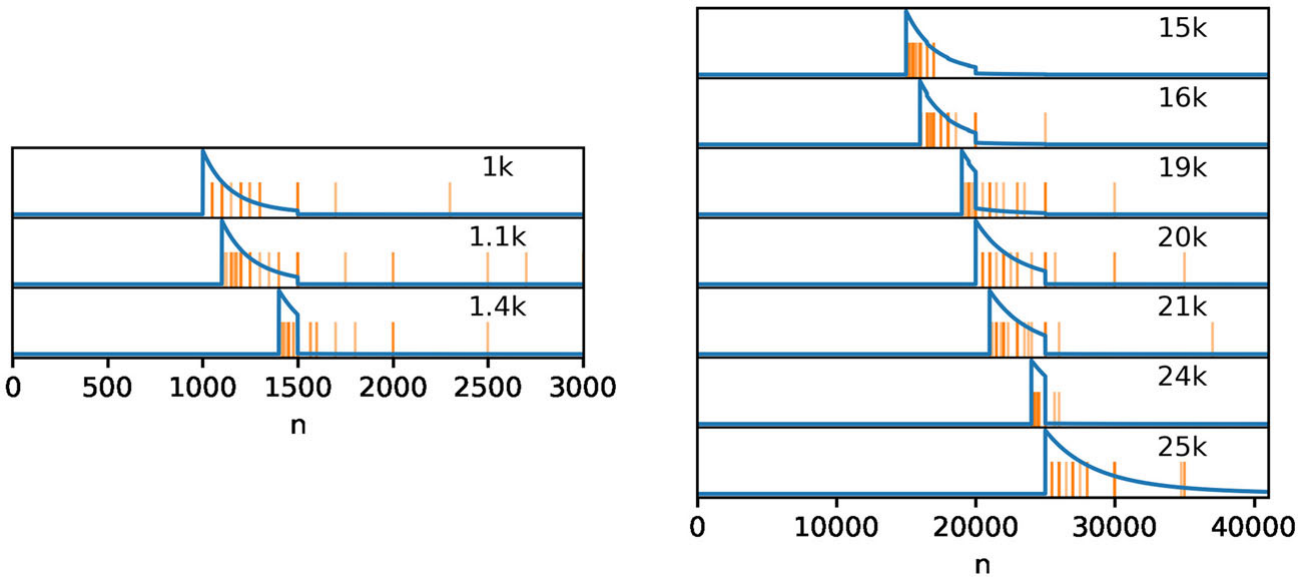
The predicted posterior over numerals after hearing “more than *n*” with a distributed zeta prior (s=7,α=0.1,β=5), for large values of *n*. Participants' guesses are shown as vertical orange lines, while the predicted distribution of the guesses is shown with a blue line. Note that the parameters are the same as in Fig. 9.

The resulting posterior for the small numerals in experiments 2 and 3 can be seen in Fig 9, along with the specific experimental observations for comparison. Similarly, Fig. 10 shows this for the large numerals in experiment 4 from Hesse and Benz (2020). The same parameters are used in the two figures, and only the involved modified numerals change. Nonetheless, the range of predicted guesses increases in a way that is similar to the one observed: while the predicted guesses in Fig. 9 are within a few dozen of the modified numeral, the ones in Fig. 10 are scaled proportionally to a few hundreds or a few thousands. Moreover, the squeezing effect also scales in a way consistent with the data: while the numerals squeeze below numbers at the roundness of 100 in Fig. 9, the same level of roundness does not produce a visible squeezing effect for the large modified numerals in Fig. 10, where instead greater roundness is needed to produce the squeezing effect. In sum, a single prior can predict the three main features of the data: first, the effect of magnitude on the guesses; second, the production of extreme values in experiment 1 without modified numerals; third, the squeezing effect in a way that scales with the modified numeral.

While the RSA model predicts the main features of the experimental data from Hesse and Benz (2020), Figs. 9 and 10 are in some respects quantitatively different from the data. Specifically, in some cases we get a *spill‐over effect* ^15.^: the guesses observed in the experiment are higher than would be expected based on the model's predictions. For instance, for n=1.4k in Fig. 10 several of the guesses are higher than expected. A possible reason for this is that the relative costs of different levels of roundness might be different from the simple one assumed in the model. Future work could fit the cost of the individual levels of granularity based on the data. Another possible reason is that the model does not account for the hierarchical structure of the data, where different contexts might be associated with different priors across participants. This can be important both because some of the contexts in experiment 4 could have lightweight priors, and, therefore, a decreasing mean excess function, and because the different heavy‐tailed contexts might have different parameters.

## 4 Conclusions

Overall, our model provided a quantitative account of some aspects of the way participants understand the modified numeral “more than *n*.” Various features of the data are predicted by our model. First, and most importantly, the general shape of the guesses is predicted to be similar to option b in Fig. 1, which is consistent with the data in Hesse and Benz (2020). Second, the model captures the patterns of implicature discussed at the beginning of the paper: the prima facie surprising lack of strong implicatures for successive numerals and the dependency of implicatures on roundness and magnitude. In particular, assuming zeta‐distributed priors for the heavy‐tailed contexts studied in experiments 3 and 4 from Hesse and Benz (2020) we can account for the increasing range of the guesses as a function of the magnitude of the modified numeral.

The work in this paper could be extended in various ways. First, a Bayesian statistical model can be developed to fit the data in Hesse and Benz (2020), and a Bayesian model comparison can be used to compare our account to the ANS account. The account proposed in Hesse and Benz (2020) is not a quantitative account of production, and, therefore, it would have to be extended to directly predict experimental data. Cummins (2022) offers further considerations on the possible relations between the interpretation of modified numerals and the ANS. Moreover, a hierarchical model could take into account the differences in priors across contexts and participants and possibly help explain the spill‐over effect.

Second, the model could be extended to include more modified numerals. For instance, Hesse and Benz (2020) also include the modified numeral “less than.” Other modified numerals that could be modeled are “at most *n*” and “at least *n*,” which have been shown to differ from “less than n+1” and “more than n−1” in interesting ways (Spector, 2020).

Third, the model could be extended to make predictions for the case of light‐tailed priors. In a simple version of the extended model with light‐tailed priors, greater values of *n* would result in guesses that are closer, rather than further, from the modified numeral. However, this assumes that participants do not update their prior based on the information provided by the modified numeral itself. This assumption might be false: the probability of a person being taller than 100 m is so low that, upon hearing that a person is taller than 100 m, a participant would most likely give up on usual world knowledge and work with a different set of assumptions.^16.^ The transition from the usual parametric assumptions to the ones adapted to the observed modified numeral could itself be studied in a Bayesian framework.

The model also makes some assumptions and predictions beyond the data in Hesse and Benz (2020) that could be tested experimentally. For instance, it assumes that listeners end up with a full posterior distribution over numbers after hearing a modified numeral. However, it is not obvious that language users would possess representations of this kind, especially over an infinite set of numbers. Moreover, a listener would in principle need to calculate implicatures over an infinite set of possible utterances, which is implausible from a processing point of view. While we see the RSA model as a computational level model, these considerations should be taken into account if the gap to the algorithmic level is to be bridged.^17.^ We leave all these possibilities to future work.

## Notes

1 For lack of conventional and apt terminology, in the following we will slightly abuse the term “implicature,” using it for phenomena that might not traditionally count as such. We will for instance speak of an “accumulation of implicatures.” The context and model will clarify what is meant in each case.

2 This assumes a contextual restriction to integers. In the following, we will keep this assumption unless explicitly stated otherwise. We thank a reviewer for pointing out this needed qualification.

3 All the distributions we consider are proper distribution, which excludes such distributions as a degenerate density over R. We think this is a reasonable default assumption as it also ensures that the listener's posterior defines a generative model.

4 As we discuss below in more detail, experimental work in Hesse and Benz (2020) shows that option *b*, with some caveats, is in fact closest to how “more than *n*” is actually interpreted.

5 Hesse and Benz (2020) give a partial characterization which we discuss below. Moreover, Benz (2015) gives an account of implicature patterns of “more than *n*” for round *n*s which is some ways similar to the one presented in this paper, but stops short of characterizing the whole listener's posterior. Cummins (2015, p. 182), for example, offers some remarks on the relation between modified numerals and probabilistic thinking.

6 Note that not all possible scales are used to determine roundness. For instance, 202 is less round than 200, despite being divisible by a number, 101, which is greater than the greatest divisor of 200, namely 100. This indicates that the scale of multiples of 101 does not play a role in determining roundness. We do not develop an account of which scales influence roundness, but rather rely on previous characterizations from the literature (see e.g., Jansen & Pollmann, 2001).

7 Cummins (2013) give a more theoretically grounded and precise account of the implicature patterns for modified numerals based on optimality theory (Prince & Smolensky, 2008). While these previous accounts include more phenomena than are discussed here, they do not provide a full characterization of p(·|MTn). Therefore, here we focus on the experimental results.

8 Probabilities are upper‐truncated at 10,000 for simplicity, and the possible signals all the expressions “more than *n*” with 0≤n≤10,000. https://github.com/thelogicalgrammar/modifiedNumeralscontains all the code needed to reproduce this and the following results.

9 Note that we mean this at the computational level. It is at this stage unclear what algorithmic story could be given for this. One possibility is that only a small set of families of distributions are used to encode priors, selected to make inference particularly simple, and only a small number of numerals are considered at each level of granularity. We thank the reviewer for pointing to this question.

10 This characterization provides an intuitive understanding, but it cannot be a definition as not all distributions have a mean. In addition, the concept of “tail” is in itself a vague concept. Nonetheless, this characterization will suffice for the present exposition.

11 The prior for the geometric family's *p* parameter is uniform in [0, 1], the prior for the zeta family's *s* parameter is a half normal distribution with sd=15, and the prior for the negative binomial's *p* and *n* parameters are uniform in [0,1] and a half normal with σ=20, respectively.

12 The function that for every element in the distribution's domain gives the probability of a random variable with the distribution taking that value.

13 Strictly speaking, the observation not only involves the mean but the whole shape of the distribution, related to the condition of *scale invariance*. However, for simplicity we only consider the mean excess function. As will become clear below, the zeta distribution also approximately satisfies the more general condition.

14 Tail behavior is in general better understood for distributions with continuous support. For instance, among these only the generalized Pareto family perfectly satisfies our condition of a linearly increasing mean function (Ghosh & Resnick, 2011). The zeta distribution can be seen as a discrete version of the Pareto distribution.

15 We thank an anonymous reviewer for suggesting this name for the effect.

16 Hyperbole is one case where utterances with extremely low probability can lead to adopting a different model. This was studied in detail in the RSA framework by Kao, Wu, Bergen, and Goodman (2014).

17 We thank an anonymous reviewer for raising these points.
